# Endogenous innate sensor NLRP3 is a key component in peritoneal macrophage dynamics required for cestode establishment

**DOI:** 10.1007/s12026-024-09496-3

**Published:** 2024-06-06

**Authors:** Irán Flores-Sotelo, Natalia Juárez, Marisol I. González, Auraamellaly Chávez, Danielle T. Vannan, Bertus Eksteen, Luis I. Terrazas, José L. Reyes

**Affiliations:** 1 Laboratorio de Inmunología Experimental y Regulación de la Inflamación Hepato-Intestinal, UBIMED, FES, Iztacala, UNAM, Tlalnepantla de Baz, Estado de México Mexico; 2 Boston Scientific Corporation, Urology Division, 200 Boston Scientific Way, Marlborough, MA USA; 3 Aspen Woods Clinic, Calgary, AB Canada; 4 Laboratorio de Inmunoparasitología, UBIMED, FES Iztacala, UNAM, Tlalnepantla de Baz, Estado de México Mexico

**Keywords:** NLRP3, *Taenia crassiceps*, Macrophages, Th2, Cestode, Interleukin 15

## Abstract

The NLRP3 receptor can assemble inflammasome platforms to trigger inflammatory responses; however, accumulating evidence suggests that it can also display anti-inflammatory properties. Here, we explored the role of nucleotide-binding oligomerization domain pyrin-containing protein 3 (NLRP3) in *Taenia crassiceps* experimental infection, which requires immune polarization into a Th2-type profile and peritoneal influx of suppressive macrophages for successful colonization. NLRP3 deficient mice (NLRP3^−/−^) were highly resistant against *T. crassiceps,* relative to wild-type (WT) mice. Resistance in NLRP3^−/−^ mice was associated with a diminished IL-4 output, high levels of IL-15, growth factor for both innate and adaptive lymphocytes, and a dramatic decrease in peritoneum-infiltrating suppressive macrophages. Also, a transcriptional analysis on bone marrow-derived macrophages exposed to *Taenia*-secreted antigens and IL-4 revealed that NLRP3^−/−^ macrophages express reduced transcripts of relm-α and PD-1 ligands, markers of alternative activation and suppressive ability, respectively. Finally, we found that the resistance displayed by NLRP3^−/−^ mice is transferred through intestinal microbiota exchange, since WT mice co-housed with NLRP3^−/−^ mice were significantly more resistant than WT animals preserving their native microbiota. Altogether, these data demonstrate that NLRP3 is a component of innate immunity required for *T. crassiceps* to establish, most likely contributing to macrophage recruitment, and controlling lymphocyte-stimulating cytokines such as IL-15.

**Supplementary Information:**

The online version contains supplementary material available at 10.1007/s12026-024-09496-3.

## Introduction

Immune cells are equipped with a variety of receptors known as pattern recognition receptors (PRRs), whose function is to activate and modulate the cellular response. In general, PRRs encompass C-type lectins (CTLs), RIG I-like receptor (RLRs), Toll-like receptors (TLRs) and NOD-like receptors (NLRs)[1]. Within the NLR family, several members are composed by proteic domains which enable them to activate caspases through interacting with intermediate adaptor molecules (e.g*.*, ASC) and ultimately proteolytically processing of inflammatory cytokines such as interleukin (IL)-1β and IL-18. These proteic platforms composed by NLRs-ASC-caspases were named inflammasomes [2] and are activated upon recognition of both pathogen-associated molecular patterns (PAMPS) and danger-associated molecular patterns (DAMPs). NLRP3 belongs to the subfamily of inflammasome-assembling NLRs, and therefore it has mostly been associated with adverse effects in inflammatory diseases where silencing NLRP3 resulted in amelioration of tissue injury. However, these receptors seem to display dual features, for instance, mice lacking NLRP3 presented enhanced alcohol-induced liver injury when compared to WT counterparts [3]. This paradox is further supported by findings that demonstrate mouse models of chemically-induced colitis [4] and immune-mediated biliary inflammation [5] have an exaggerated immune response in the absence of NLRP3, suggesting that NLRP3 also displays tissue-protective functions. In line with this, Chung et al*.* recently reported that absent in melanoma (AIM) 2, another inflammasome-activating receptor, is indeed required to down-modulate the intensity of inflammatory reactions in the mouse kidney [6]. Thus, growing evidence shows that these inflammasome-assembling sensors can display diverse, often contrasting immune roles.

The role of NLRP3 in recognizing and responding to viral infections [7], intracellular bacteria [8, 9], and protozoans [10] has been largely explored, whereas a few studies have revealed the contribution of NLRP3 in helminth-induced infections. Parasitic helminths create a unique microenvironment where Th2-type responses and regulatory circuits co-exist, and these complex networks simultaneously allow for host protection (tissue repairing) and worm expulsion [11]. In fact, there seems to be an intriguing relationship between helminthic parasites and NLRP3 inflammasome. For example, a recombinantly-expressed cathelicidin-like peptide from the fluke *Fasciola hepatica* prevented NLRP3 activation elicited by silica particles [12], whereas a secreted cathepsin from the *Fasciola hepatica* larval stage activated NLRP3 in mouse dendritic cells (DCs) [13]. Furthermore, in *Schistosoma mansoni* experimental infection as well as DCs in vitro exposed to S*chistosoma* egg antigen (SEA), the activation of several inflammasomes (NLRP3, NLRP6, and AIM2) has been documented [14, 15], and liver pathology elicited by this fluke is largely attributed to NLRP3 inflammasome activation [16, 17]. In addition to by-stander injury elicited in helminthic experimental infections, the presence of NLRP3 modulates disease outcomes as gauged by increased resistance in mouse lacking NLRP3 infected with nematodes such as *Trichuris muris* [18] and *Nippostrongylus brasiliensis* [19]. In contrast, increased susceptibility was observed in NLRP3 deficient mice infected with *Trichinella spiralis* [20]. Hence, NLRP3 appears to be an important regulator in helminthic infections and consequently, helminth-induced infection models represent a valuable tool to explore the role of NLRP3 in immune-regulatory microenvironments. Thus, we employed the experimental infection with the larval stage of the cestode *Taenia crassiceps*. At the adult stage, this parasite naturally resides in the small intestine of canids (i.e., foxes and dogs), whereas metacestodes (larvae) dwell in the pleural and peritoneal cavity of rodent animals [21]. When rodents are experimentally infected with metacestodes of *T. crassiceps*, this offers a unique model to explore immune responses in the peritoneal cavity since these metacestodes are non-migrating forms.

Here, we report that in absence of NLRP3, mice acquired a highly resistant phenotype against *T. crassiceps* infection, which can be partly explained by their inability to expand suppressive macrophages, and the resistance can be transferred via intestinal microbiota, as co-housing experiments suggested.

## Materials and methods

### Mice and infection

Eight-week-old female wild type (WT) and NLRP3 deficient mice (NLRP3^−/−^) (mouse colony kindly donated by Dr. Daniel Muruve, University of Calgary, AB, Canada), both in C57BL/6 genetic background were used in this study. Animal colonies are bred and maintained at our mouse facilities and the use of these colonies was approved by the Bioethics Committee (CE/FESI/042022/1513) and met the recommended guidelines issued by government for experimental animal care (NOM-062-ZOO-1999). The ORF strain of *Taenia crassiceps* is maintained by serial passages in female BALB/c mice and upon 8 weeks of infection, parasites are harvested from the peritoneal cavity, washed, and counted. Experimental groups were intraperitoneally (ip.) infected with 20 non-budding metacestodes, and resistance was assessed by means of parasite counts at 4 and 8 weeks of infection.

### Blood samples

To determine the circulating profile of cytokines, blood samples were collected by conducting tail snips on experimental mice at indicated times. The obtained blood samples were collected in sterile plastic tubes containing 40 µl of anticoagulant (0.5 mM EDTA), which were next centrifuged (3500 rpm/10 min), and plasma samples were maintained at − 80 °C until used for ELISA assays.

### Cell culture

Mesenteric lymph nodes (MLN) and spleen were excised from experimental groups at sacrifice and passed through sterile 70-µM-pore nylon mesh cell strainers (Falcon, USA). Red blood cells (RBCs) were depleted with ammonium chloride buffer, and leukocyte suspensions were seeded at density of 3 × 10^6^/well in flat bottom 24-well plates (Corning Inc. Costar, USA). Cells were stimulated with 5 µg of the mitogen concanavalin A (Sigma USA) for 48 h. Cell culture supernatants were collected and stored at − 80 °C until used for ELISA assays.

### ELISA

The levels of IL-1β, IL-4 and IL-15 (Peprotech USA), and IL-18 (MyBiosource, USA) in plasma samples, and culture supernatants were determined following manufacturer´s instructions.

### Flow cytometry

When indicated, cells from both experimental groups were recovered by means of peritoneal lavage and maintained in buffer 1 (sterile 1 × PBS supplemented with FBS 10% and 2 mM EDTA, Cytiva, USA). Peritoneal cells were counted and adjusted at 1 × 10^6^ cells/ml and transferred into flow cytometry buffer (PBS with 1% FBS and 0.05% sodium azide, Amresco, USA) in order to proceed for staining. Cell samples were incubated 30 min. at 4 °C with the combinations of fluorochrome-conjugated antibodies as follows: suppressive macrophages (PECy7-F480, PE-PDL1, and APC-PDL2), eosinophils (PECy7-F480 and APC-Siglec F), and B regs (APC-CD19, PE-PDL1 and BV421-PDL2). Samples were acquired in Attune® NXT and analyzed with FlowJo V10 software (USA), representative plots gated on singlets, and live cells are shown.

### Bone marrow macrophage stimulation

Bone marrow precursors were flushed from femur and tibias from 8-week-old WT and NLRP3^−/−^ mice and differentiated into mature macrophages by incubating them in presence of M-CSF (20 ng/ml, Tonbo Bioscience, USA) for 7 days in RPMI 1640 cell culture medium (Biowest, France) supplemented with 20% FBS (Cytiva, USA) and antibiotics (Pen/Strep, Biowest, France). On days 2 and 4, fresh medium containing M-CSF was added. Mature macrophages were harvested by incubating them in Hank’s balanced solution containing EDTA (1 mM) and TrypLE Express (Gibco, USA) (1:1 solution) for 30 min at 37 °C and adjusted to 5 × 10^5^ cells/ml and seeded into 24-well plates (Corning Inc. Costar, USA). Cells were left untreated as control and experimental groups were incubated with IL-4 (20 ng, Peprotech USA), *Taenia crassiceps* excreted-secreted antigens (TcES, 50 µg) and the combination of both stimuli (TcES/IL-4). Total RNA was extracted from experimental groups at 24- and 48-h post-incubation, and it was used as template for cDNA synthesis (Revert Aid First Strand cDNA Kit, Thermo Scientific, USA). Once cDNA was synthesized, samples were prepared for PCR reactions and indicated gene products (see suppl. Table 1) were visualized in 1.5% agarose gel.

### Peritoneal macrophages from TcES/IL-4-injected mice

For ex vivo analysis, both WT and NLRP3^−/−^ mice were ip. injected with the combination of IL-4 (20 ng, Peprotech, USA) and TcES (50 µg) in a final volume of 500 µl. Since macrophages are predominant in 48–72 h peritoneal infiltrates [22], peritoneal lavage was carried out at 72 h after ip. injections, and total cells were counted, and 1 × 10^6^ cells were stained to determine the presence of membrane-bound PDL1 and PDL2 on macrophages, as described in the “Flow cytometry” section.

### Co housing-experiment

Four-week-old WT and NLRP3^−/−^ mice were housed in separate cages as control groups, whereas animals from the same batch were co-housed, 2 animals from each strain per cage for 4 weeks. At 8 weeks of age, co-housed animals were separated and transferred into new cages. Animals from control and co-housed groups, were ip. infected with 20 metacestodes and sacrificed 8 weeks post-infection where parasites were enumerated, and flow cytometry and cell cultures were carried out, as previously described.

### Data presentation

Comparisons between WT and NLRP3^−/−^ mice were analyzed by unpaired student’s *t* test with *Welch’s* correction, where 95% confidence interval and *p* < 0.05 was accepted as significantly different. Multiple comparisons analysis was conducted by using one-way ANOVA followed by post hoc Tukey’s test in the Graphpad Prism V9 software (USA).

## Results

### Mice lacking NLRP3 show an altered composition of peritoneal immune cells and are resistant to *T. crassiceps* infection.

**Fig. 1:**
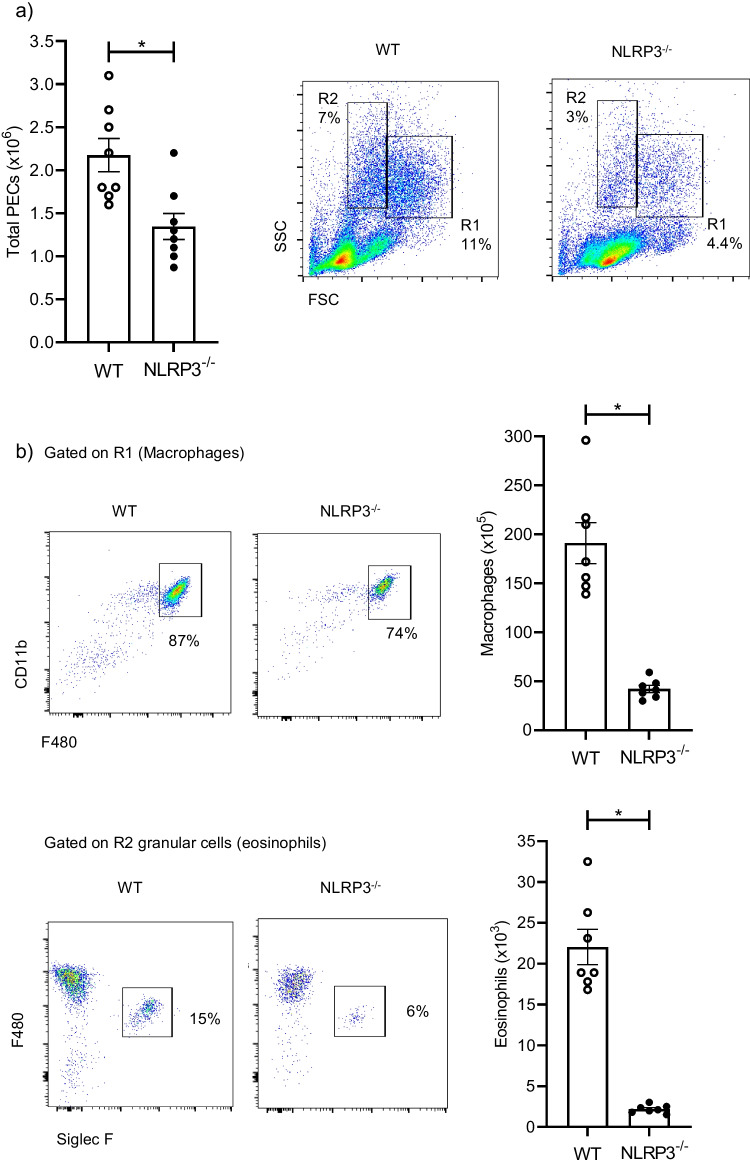
NLRP3 is involved in maintaining homeostatic distribution of peritoneal cells. Age- and sex-matched uninfected WT and NLRP3^−/−^ mice were sacrificed, and peritoneal cells were retrieved and counted to characterize immune cell populations. Panel **a** shows the differences found in terms of absolute numbers of peritoneal exudate cells (PECs) between WT and NLRP3^−/−^ groups, and representative plots are shown, where gates of monocytic (R1) and granular cells (R2) are evident. Next, cells were prepared for staining and representative plots and percentages of macrophages (F480^+^ CD11b^+^) and eosinophils (F480^lo^ Siglec F^+^) are shown in (**b**), also absolute numbers of both populations are depicted in graphs. Data shown are mean ± SEM from 3 experiments (*n* = 8 each group) where each point represents an individual mouse, and * indicates *p* < 0.05 when compared with unpaired student’s *t* test

Since *T. crassiceps* dwells in the peritoneal cavity, waves of both innate and adaptive immune cells are constantly recruited to this site. First, we determined total cell counts and immune cell populations assayed by flow cytometry. Surprisingly, the number of resident peritoneal cells was different between WT and NLRP3^−/−^ mice before infection. We harvested resident total peritoneal cells from WT animals and found an average of 2 × 10^6^ peritoneal exudate cells (PECs), whereas NLRP3^−/−^ mice presented a significant reduction ~ 30% with an average of 1.4 × 10^6^ PECs (Fig. 1a). Moreover, when cells were immuno-typed, flow cytometry assays revealed that reduced populations included macrophages and eosinophils (Fig. 1b), although other immune cell types may also be underrepresented. This suggests an intrinsic defect in peritoneal homeostasis in female NLRP3^−/−^ mice. Next, we proceeded to infect both WT and NLRP3^−/−^ mice with metacestodes harvested from 8-week-infected BALB/c mice.

**Fig. 2:**
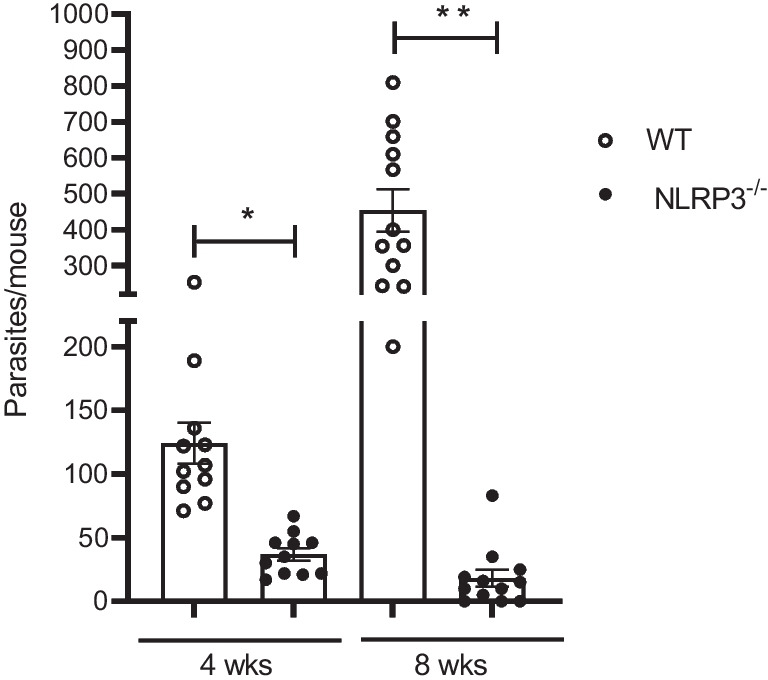
*T. crassiceps* parasites require the NLRP3 receptor to successfully colonize its host. Experimental mice from both groups (WT and NLRP3^−/−^) were infected with 20 metacestodes and susceptibility was assessed at 4 and 8 weeks upon infection. Average of parasites per mouse recovered from WT and NLRP3^−/−^ mice is shown, where lack of NLRP3 turned mice into highly resistant. Values represent mean ± SEM from 3 experiments (*n* = 11–12 each group) where * is *p* < 0.05, and ** is *p* < 0.01 when unpaired student’s *t* test was performed

The experimental infection with *T. crassiceps* results in chronic persistence of this parasite in its host peritoneal cavity. To achieve this latter, *T. crassiceps* progressively creates an environment dominated by a Th2 response [23, 24] and modulates antigen-presenting cells (APCs) like macrophages [25]. Therefore, both the emergence of Th2 cytokines and alternatively activated macrophages (AAMs) dictate the disease outcome. In this study, we observed that infected WT mice presented an increasing number of parasites determined at weeks 4 and 8 post-infection (121 ± 7.8 and 477 ± 14.5 parasites/mouse, respectively) (Fig. 2). Conversely, similarly infected NLRP3^−/−^ mice were highly resistant as evidenced for significantly reduced numbers of parasites at 4 and 8 weeks of infection (30 ± 3.6 and 10 ± 3.5 parasites/mouse, respectively) (Fig. 2). Thus, endogenous NLRP3 turned out to be indispensable for *T. crassiceps* establishment.

**Fig. 3:**
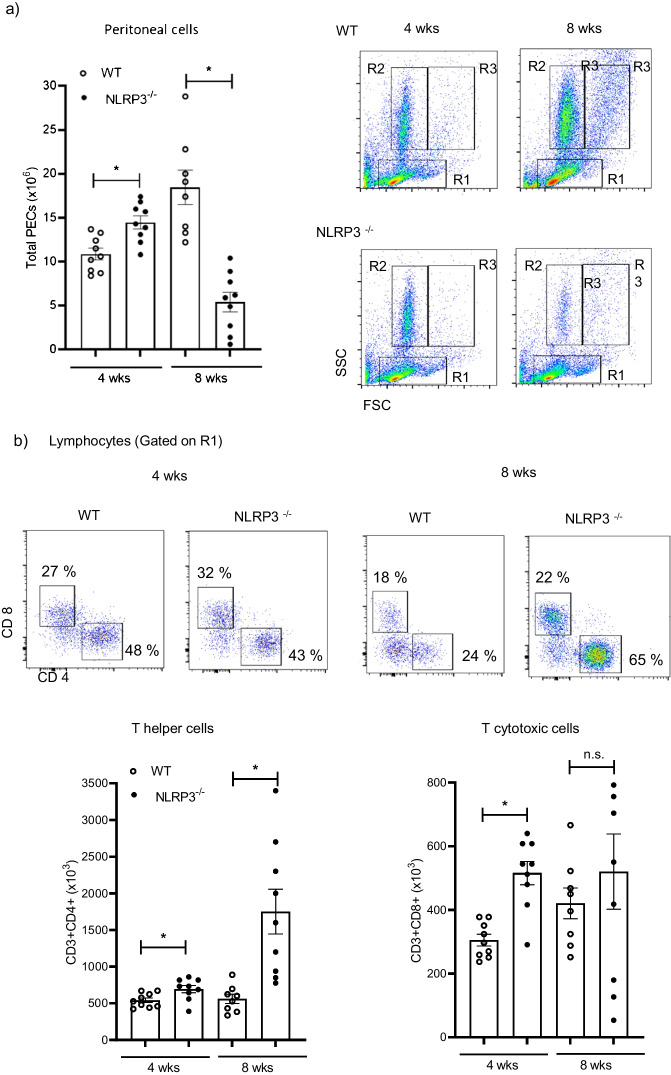

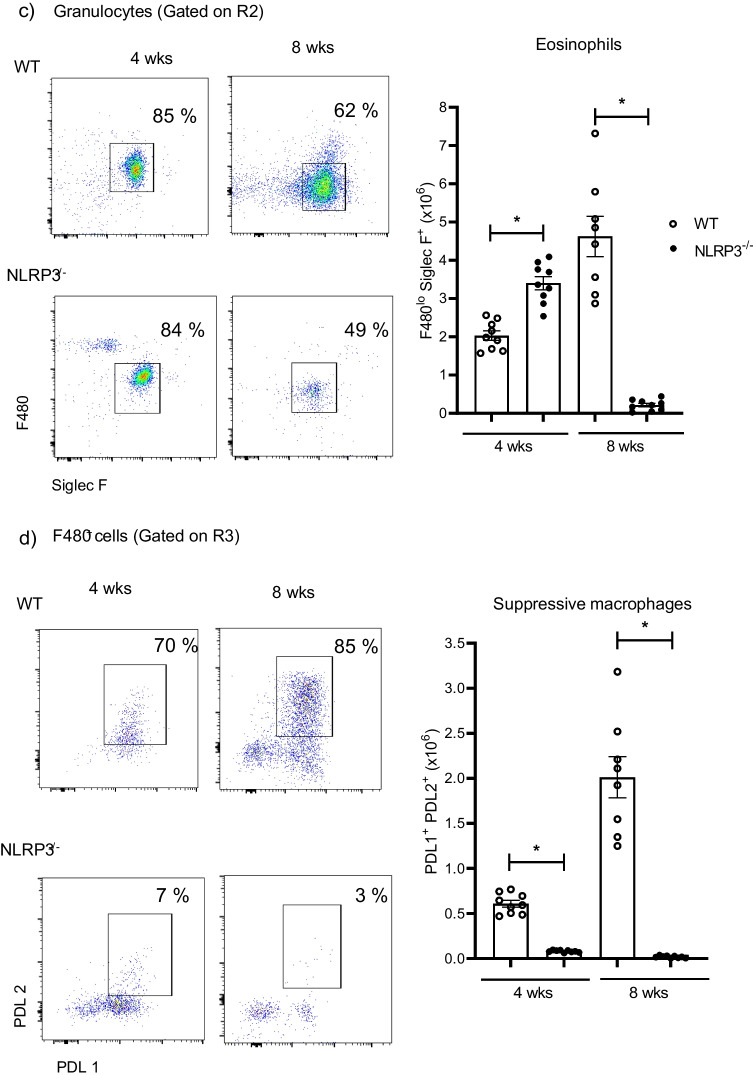
Immunophenotyping of peritoneal cells. At 4 and 8 weeks of infection, a peritoneal lavage and flow cytometry were carried out on experimental mice to identify immune cell populations. Collected cells were acquired in ATTUNE® cytometer where singlets and live cells were included in the analysis. Panel a shows the absolute numbers of peritoneal cells found at indicated times of infection and cell distribution based on FSC and SSC parameters. In b, cells selected from lymphocyte gate (R1) were further selected from T cell population CD3^+^CD19^−^ cells (data not shown) and assayed for CD4 and CD8, where resistant NLRP3^−/−^ mice presented an abundant population of CD4^+^ T cells. Also, absolute numbers of lymphocytes show a significant increase of T cells in peritoneal cavity of NLRP3^−/−^ mice as compared to WT individuals. Panel c shows representative plots with indicated percentages and absolute numbers of eosinophils (F480^lo^ Siglec F^+^) gated on R2 (granular cells), where a few eosinophils remained in peritoneal cavity of resistant NLRP3^−/−^ mice. Interestingly, F480 fluorescence is decreased on cells from week 8 as compared to cells from week 4. In panel d, the percentage of macrophages (gated from F480^+^ cells) expressing both PDL1 and PDL2 in experimental groups is shown. WT mice progressively increased influx of suppressive macrophages, whereas NLRP3^−/−^ mice presented only a few macrophages which mostly express PDL1 only. Representative dot plots and analysis of absolute numbers are from 3 experiments (*n* = 8–9 per group) mean ± SEM is shown, where * indicates *p* < 0.05 when unpaired student’s *t* test was carried out

Upon infection, peritoneal cells progressively increased in samples obtained from WT animals, in contrast, peritoneal cells in NLRP3^−/−^ individuals peaked at 4 weeks of infection and then declined reaching a substantial reduction on week 8 (Fig. 3a). Flow cytometry assays revealed that based on FSC and SSC parameters, peritoneal cells from infected animals could be gated on regions compatible with lymphocytes (R1), granular cells (R2), and monocytes/macrophages (R3) (Fig. 3a). Interestingly, when lymphocytes were characterized, it was evident that the presence of *T. crassiceps* in WT mice caused lower numbers of both CD4^+^ and CD8^+^ T cells (gated on CD3^+^ CD19^−^) (Fig. 3b). In contrast, we observed that concomitant to parasite clearance in NLRP3^−/−^ mice, higher numbers of T lymphocytes remained in the peritoneal cavity (Fig. 3b). This is suggestive that, the well-known hypo-proliferative response caused by *T. crassiceps* as immune evasion strategy [26] is not occurring in NLRP3^−/−^ mice.

Additionally, helminthic infections trigger eosinophil expansion, and we sought evidence of any significant changes within this population. Cells gated on R2 (granulocytes) demonstrated to be predominantly eosinophils (> 80% were F480^lo^ Siglec F^+^) in infected animals as compared to a negligible eosinophil population found in uninfected mice from both experimental groups (Fig. 1b). A massive increase in peritoneal eosinophils was evident as early as 4 weeks of infection, where despite of similar percent of eosinophils (shown in plots), absolute numbers were higher in NLRP3^−/−^ mice than those numbers found in *Taenia*-infected WT mice (Fig. 3c). Upon 8 weeks of infection an abundant population of eosinophils was observed within peritoneal cavity of WT mice, where parasites were still present, in contrast, in NLRP3^−/−^ mice a significantly reduced population of eosinophils was found (Fig. 3c). An intriguing finding was the fact that eosinophils recovered from WT as well as NLRP3^−/−^ mice (gated from the same region) at 8 weeks of infection displayed a reduced expression of the antigen F480 (Fig. 3c). We assayed these cells for Ly6G marker to rule out neutrophil presence, given neutrophils also express Siglec F [27], and they preserved eosinophil identity as only 1–2% of cells were Siglec F^+^ neutrophils as gauged by Ly6G co-expression (data not shown). Thus, whereas *T. crassiceps* infection in WT mice caused a progressive increase of peritoneal eosinophils, similarly infected NLRP3^−/−^ counterparts, which cleared the infection, presented lower numbers of eosinophils.

We have shown that recruitment and re-programming of macrophages is central in allowing *T. crassiceps* growth in the peritoneal cavity of experimentally infected mice [28]. Thus, we aimed to identify PDL1^+^ PDL2^+^ macrophages in peritoneal cavity and observed that the majority of peritoneal macrophages harvested at 4 weeks of infection from infected WT mice were PDL1^+^ PDL2^+^ (70% from F480^+^ cells). This macrophage influx was further increased at 8 weeks of infection, where PDL1^+^ PDL2^+^ macrophages reached 85% of total macrophages; interestingly, other macrophage populations (i.e., double negative and PDL1 single positive) were also found, which suggested a steady infiltration of macrophages (Fig. 3d). Interestingly, NLRP3^−/−^ mice showed mostly PDL1 single positive macrophages and a significantly reduced population of PDL1^+^ PDL2^+^ peritoneal macrophages (~ 7% from F480^+^ cells) at 4 weeks of infection. At 8 weeks of infection, NLRP3^−/−^ mice exhibited a nearly absent population of these macrophages (Fig. 3d). Therefore, the inability to recruit and likely re-program macrophages might explain the resistance displayed by NLRP3^−/−^ mice against *T. crassiceps* infection, which ultimately results in optimal T cell responses and reduced eosinophils resulting from parasite clearance.

### NLRP3 is required for optimal Th2 polarization in *T. crassiceps* infection

**Fig. 4:**
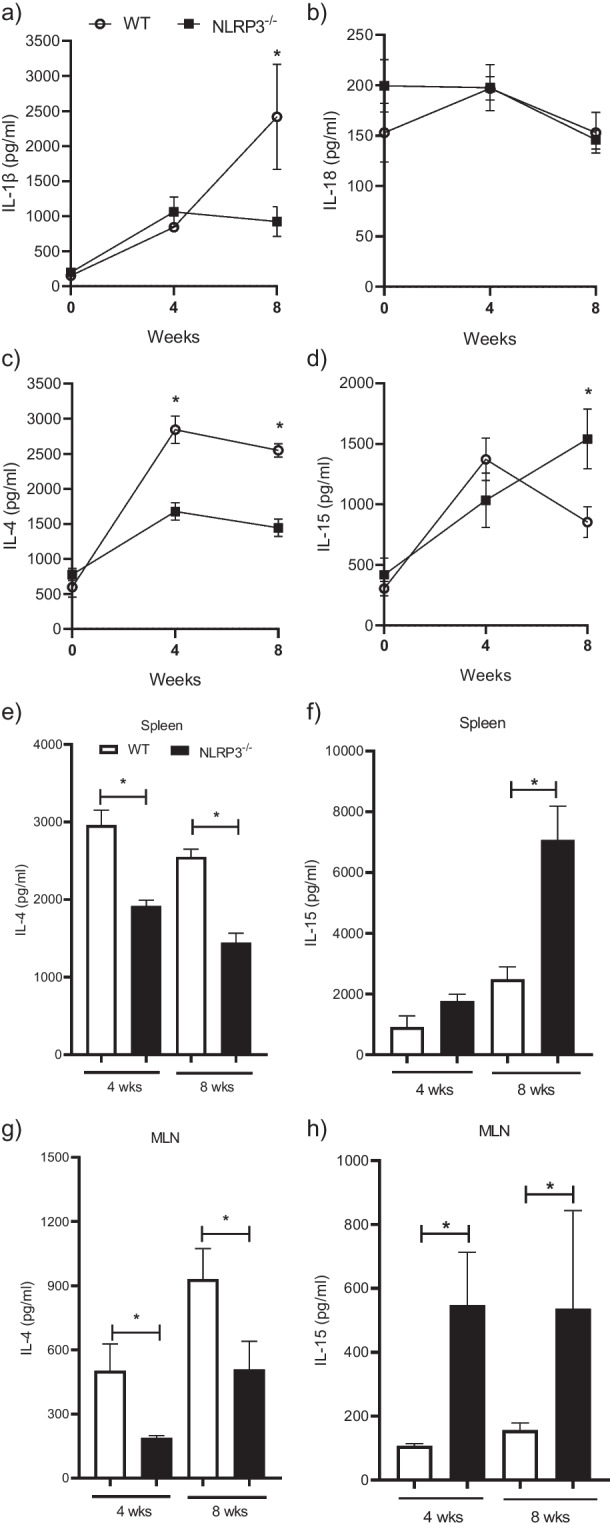
Mice lacking NLRP3 presented a diminished Th2 response. Cytokine output was quantified in plasma samples from experimental animals. Panels (**a**) and (**b**) show the main products of NLRP3 inflammasome, IL-1β and IL-18. Circulating levels of IL-4, as indicative of Th2 response, are depicted in (**c**), while panel (**d**) shows the differences of IL-15 levels, which is a survival factor for lymphocytes. Panels (**e**), (**f**), (**g**), and (**h**) show cytokine levels in supernatants from spleen and MLN cell culture supernatants as indicators of systemic and local responses. Data are from 3 experiments (*n* = 8–9 each group) where mean ± SEM is shown and * is *p* < 0.05

We aimed to identify cytokine profiles in plasma samples to determine the influence of NLRP3 on cytokine production. First, we quantified IL-1β and IL-18 as the main products of canonical NLRP3 activation and observed that IL-1β showed an increasing pattern as disease progressed in WT animals reaching significantly higher levels in WT than in their NLRP3^−/−^ counterparts only at 8 weeks of infection (2417 ± 752 pg/ml and 953 ± 242 pg/ml, respectively) (Fig. 4a). Interestingly, both experimental groups presented negligible circulating levels of IL-18 with a decreasing trend, and no differences were found between WT and NLRP3^−/−^ mice infected with *T. crassiceps* during 8 weeks of follow-up (Fig. 4b). On the other hand, early reports showed that a switch from Th1 to Th2 immune response is imperative during *T. crassiceps* infection [24], and high systemic IL-4 levels are indicative of successful colonization by *T. crassiceps* parasites. Thus, we quantified IL-4 in plasma samples and found that *T. crassiceps* infection in WT mice elicited an increased and sustained IL-4 production as compared to uninfected animals (Fig. 4c), whereas in infected NLRP3^−/−^ mice significantly reduced IL-4 levels were observed when compared to those found in WT animals (Fig. 4c), suggesting that NLRP3 is involved in Th2 polarization in *T. crassiceps* infection. Accordingly, IL-4 release was also found to be decreased in secondary lymphoid organs from infected NLRP3^−/−^ mice when compared to IL-4 levels produced by mitogen-activated cells from WT animals (Fig. 4e and g).

Additionally, it has been reported that IL-15 is a cytokine overproduced by NLRP3^−/−^ mice induced with intestinal inflammation [4] and a likely role for IL-15 in *T. crassiceps* infection has not been explored. Hence, we determined circulating IL-15 levels and observed that during the chronic stage of disease (8 weeks of infection) resistant NLRP3^−/−^ mice produced higher levels of this lymphocyte growth factor, as compared to those found in WT mice (1540 ± 240 pg/mL versus 853 ± 130 pg/ml, respectively) (Fig. 4d), indeed, IL-15 levels were higher in NLRP3^−/−^ mice starting on week 6 (data not shown). Interestingly, mitogen-activated MLN cells showed a steady increased IL-15 levels starting on week 4 of infection in NLRP3^−/−^ mice, whereas spleen levels were higher only at the chronic stage of infection (Fig. 4). These data suggest that IL-15 may also be a target for *T. crassiceps*, and its supression is part of the immune-regulatory strategy.

### The presence of NLRP3 enables transcriptional activity of suppressive ligands in BMMs

**Fig. 5:**
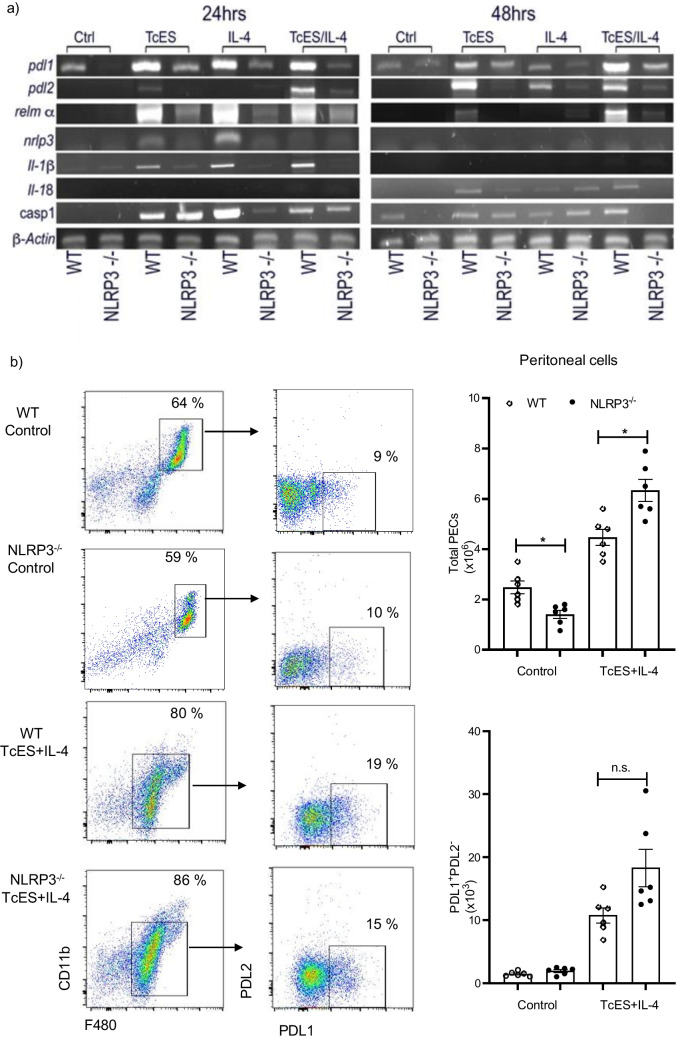
NLRP3 transcriptionally activates a suppressor phenotype in macrophages. Mature macrophages were obtained by incubating bone marrow precursors in the presence of M-CSF, and these cells seeded at density of 0.5 × 10^6^/well were incubated with TcES (50 µg) ± IL-4 (20 ng). RNA extraction was carried out at 24 and 48 h of incubation and transcripts for markers of suppressive ability (*pdl1* and *pdl2*) and alternative activation (*relm α*) were determined, representative agarose gel image is shown in panel (**a**) (*n* = 4, from 1 experiment). Additionally, WT and NLRP3^−/−^ mice were *ip.* injected with TcES (50 µg) plus IL-4 (20 ng), and peritoneal cells retrieved and stained with fluorochrome conjugated PDL1 and PDL2. Panel (**b**) shows representative plots with percentages of flow cytometry assays. Additionally, absolute numbers of indicated cells were retrieved from singlets and live cells analyzed from respective gates. Data are mean ± SEM from 2 experiments (*n* = 6) with similar results, where absolute numbers of indicated cells from WT and NLRP3^−/−^ mice were compared with unpaired student’s *t* test

Macrophages and DCs represent the most efficient type of APCs and helminth parasites have co-evolved to manipulate these innate cells in their mammalian hosts which ultimately also shape the adaptive immune response. In this context, *T. crassiceps* parasites create a peritoneal environment where macrophages are induced to express membrane-bound suppressive molecules such as PDL1 and PDL2 [25]. However, the signaling pathways controlling the expression of PDLs are not known, and we hypothesized that NLRP3 may be contributing to their expression. To test this, we incubated BMMs, given these are a major source of macrophages to replenish peritoneal cavity in response to infection [29], in the presence of TcES ± recombinant IL-4 for 48 h. We noticed a constitutive expression of *pdl1* in macrophages growth from WT mice, whereas in the absence of NLRP3, no signal was detected (Fig. 5a). Furthermore, *pdl1* expression was strongly induced by simultaneous exposure to TcES and IL-4 at 24 h of incubation, in contrast, a weak induction was observed in macrophages derived from NLRP3^−/−^ animals (Fig. 5a). Interestingly, upon 24 h of incubation, *pdl2* was only transcriptionally induced when using combination of TcES + IL-4 but not separately (Fig. 5a). At 48 h of incubation, *pdl2* was significantly induced in WT macrophages co-treated with TcES + IL-4, but we did not observe transcripts of *pdl2* when BMMs from NLRP3^−/−^ mice were used. Further, resistin-like molecule alpha (relm α) expression is indicative of alternative activation in macrophages, since it is induced by IL-4 signaling [29], thus we determined whether *relm α* expression is dependent on NLRP3 as well. We found that BMMs exposed to TcES and TcES/IL-4 for 24 h presented a strong induction of *relm α* gene; however, in BMMs lacking NLRP3, the expression of *relm α* was poorly induced (Fig. 5a). Also, IL-4 alone induced *relm α* expression, and this was not dependent on NLRP3. At 48 h of incubation, similar to *pdl1*, *relm α* expression decreased as compared to 24 h, and only the combination TcES/IL-4 sustained its expression (Fig. 5a). Interestingly, when macrophages were exposed to IL-4, we observed transcriptional activity of *pdl1* gene. Although this finding requires a deeper understanding, others have reported that IL-4 induced PDL1 in a particular subtype of AAMs, the multinucleated giant cells induced by biomaterials [30] as well as in CSF-1 primed bone marrow precursors from C57BL/6 [31]. Thus, in our in vitro system, NLRP3 turned out to be required to induce transcription of markers associated with suppressive/alternative programs in BMMs.

We also aimed to determine mRNA transcripts of the NLRP3 inflammasome components and interestingly, at 24-h post-incubation a low transcriptional activity was found for *nlrp3* gene in response TcES (Fig. 5a). Whereas *il-1b* gene was virtually non-activated at time points assayed by either stimulus. Of note, transcription of *il-18* gene was triggered by the stimuli used and required NLRP3, in contrast, caspase 1 (*casp1*) was induced when BMMs were exposed to TcES and NLRP3 was not required, whereas IL-4 strongly induced *casp1* which intriguingly required NLRP3.

We decided to confirm these findings and injected (ip.) the most effective combination of stimuli (TcES/IL-4) to determine the presence of PDL1 and PDL2 on cell membrane of peritoneal macrophages harvested at 72 h. As seen in Fig. 5 b, a small fraction of resident F480^+^ cells were PDL1^+^, and no significant differences were observed between control WT (3% from F480^+^ cells) and NLRP3^−/−^ mice (5% from F480^+^ cells). In response to TcES/IL-4 injection, increased numbers of F480^+^ cells were found in both groups as compared to control animals; interestingly, NLRP3^−/−^ mice recruited more macrophages than WT animals (Fig. 5b). However, peritoneal macrophages expressed PDL1 but not PDL2, and comparable levels of PDL1^+^ macrophages were found in both WT and NLRP3^−/−^ mice (18 ± 4 versus 20 ± 3, respectively), suggesting that, unlike BMMs where the activity of only one cell type is being tested, peritoneal cavity is highly complex and interactions with other cells might be affecting the induction of PDLs.

### Protection against *T. crassiceps* can partly be acquired by co-housing with resistant mice

**Fig. 6:**
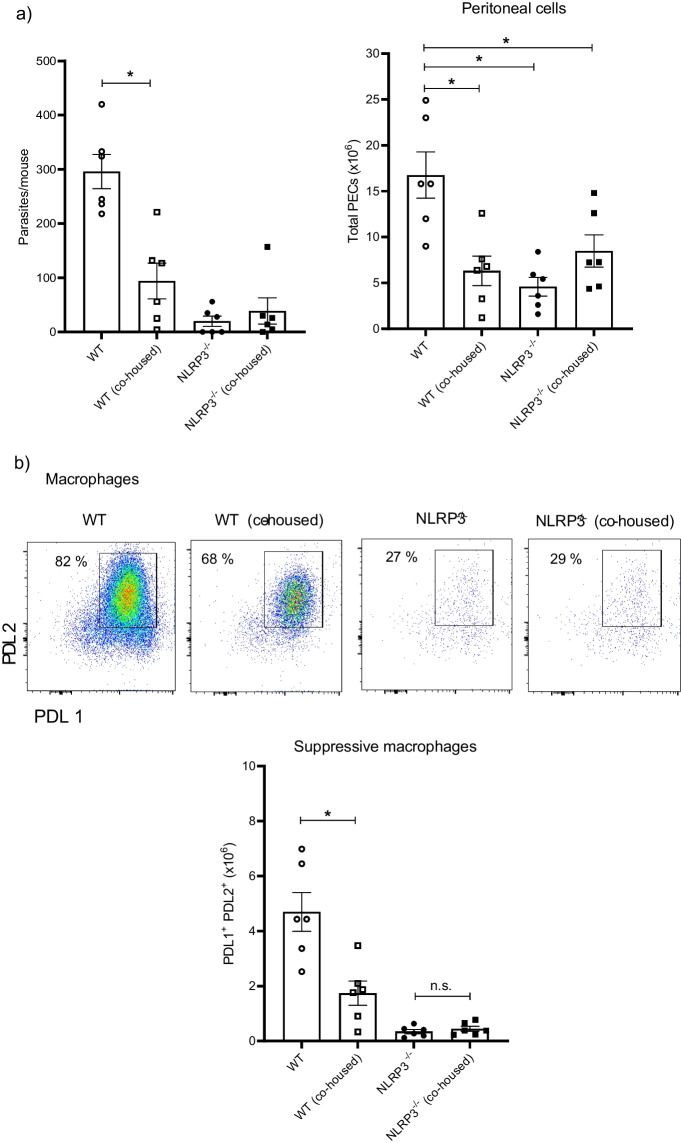

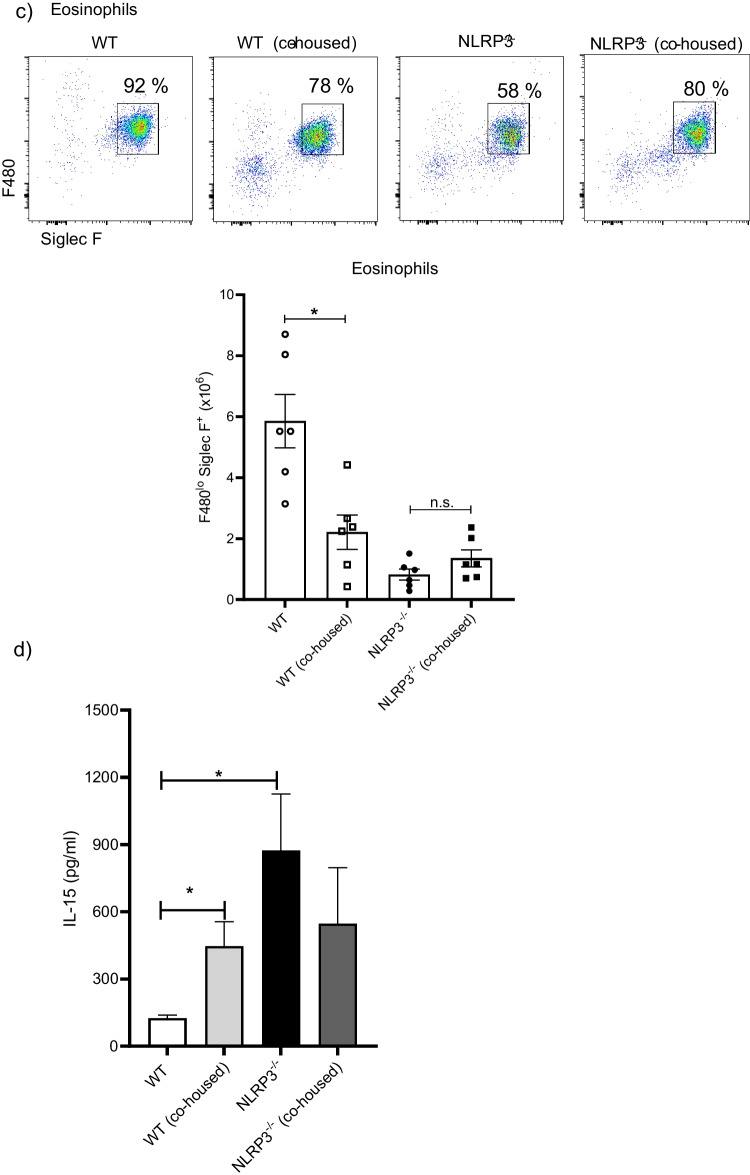
Co-housing reproduces the resistant profile found in NLRP3^−/−^ mice. In order to explore whether intestinal microbiota played a role in immune response against *T. crassiceps*, mice were co-housed prior to infection. We compared parasite numbers at 8 weeks post-infection between control groups (animals with native microbiota maintained in separated cages) and similarly infected co-housed animals. As shown in panel **a**, parasite counts evidenced that resistance to *T. crassiceps* might be transferred via intestinal microbiota, where influx of peritoneal ells was also altered by cohousing, as shown by decreased absolute numbers of peritoneal cells in WT (co-housed) group. Moreover, changes in peritoneal populations PDL1^+^ and PDL2^+^ macrophages (**b**) as well as eosinophils (**c**) resulted from co-housing experiments. Interestingly, when IL-15 levels were determined in plasma samples (**d**), WT (co-housed) animals, which displayed enhanced resistance to *T. crasscieps* infection, when compared to WT control mice, also showed increased IL-15 production. Data are mean ± SEM from 2 experiments with similar results (*n* = 6 each group), where significant differences (*p* < 0.05) are indicated as * when groups were compared with ANOVA and multiple comparisons

Intestinal microbiota shapes the immune response throughout lifetime and is therefore involved in maintaining homeostasis as well as in promoting pathological conditions when dysbiosis is developed. We tested whether co-housing of WT and NLRP3^−/−^ could phenocopy the resistance against *T. crassiceps*. Interestingly, WT mice co-housed with NLRP3^−/−^ mice displayed an enhanced resistance when compared to WT mice maintained in separated cages harboring their native microbiota. Parasite counts revealed that WT control animals harbored, at 8 weeks of infection, in average 271 ± 31 metacestodes, whereas in WT mice co-housed with NLRP3^−/−^ animals, an average of 94 ± 33 metacestodes per mouse were recovered (Fig. 6a), showing that WT mice co-housed with NLRP3^−/−^ mice for 4 weeks enhanced their resistance against *T. crassiceps* infection. On the other hand, control NLRP3^−/−^ mice were consistently resistant as evidenced with low numbers of parasites found (18 ± 8 metacestodes), and we found a non-significant increase in number of parasites (34 ± 15 metacestodes) in NLRP3^−/−^ mice co-housed with WT mice (Fig. 6a). These observations support a likely role for intestinal microbiota in contributing to protection against *T. crassiceps* infection.

We next looked for changes in the distribution of peritoneal cells (macrophages and eosinophils) resulting from co-housing experiments. WT control mice had an abundant population of macrophages PDL1^+^ PDL2^+^ relative to WT mice co-housed with NLRP3^−/−^ littermates (Fig. 6b). As expected, control NLRP3^−/−^ mice presented a remnant population of PDL1^+^ PDL2^+^ macrophages, which was negligibly affected in NLRP3^−/−^ mice co-housed with WT individuals (Fig. 6b). On the other hand, infected WT control mice presented evident peritoneal eosinophilia and surprisingly, this population was found to be significantly reduced in WT animals co-housed with resistant NLRP3^−/−^ mice (Fig. 6c). Both NLRP3^−/−^ control as well as NLRP3^−/−^ co-housed mice showed no significant changes in parasite numbers and suppressive macrophages; however, NLRP3^−/−^ mice co-housed with WT animals presented increased numbers of eosinophils (Fig. 6c). These data suggest that early-life co-housing for 4 weeks is enough to partly transfer resistance against this non-intestinal tapeworm; interestingly, a dramatic impact of microbiota exchange is only observed in WT mice when compared to changes acquired by NLRP3^−/−^ mice.

## Discussion

NLRP3 activation is triggered during pathogen invasion and upon sensing of danger-associated signals, thereby, NLRP3 is a driver of early inflammatory responses by assembling inflammasome platforms involved in proteolytic processing of cytokines. It is thought that NLRP3 largely contributes to chronic Type 1 (Th1-mediated) and Type 3 (Th17-mediated) inflammatory diseases; however, NLRP3 also promotes Th2 polarization [32] as well as Th2-mediated disease such as asthma [33], showing that NLRP3 activation regulates diverse types of immunity, displaying a high plasticity which we are far from fully understanding. Here, we explored the role of NLRP3 in the context of helminth-evoked Th2/regulatory microenvironment.

Prior to infection, we observed that NLRP3^−/−^ mice presented a significant decrease in resident macrophages and eosinophils (Fig. 1a), key innate cells in helminthic infections, showing that NLRP3 is somehow required to collaborate in either cell maturation or in driving myeloid cells influx into peritoneum under homeostatic conditions. Also, whether this phenomenon is facilities-dependent cannot be ruled out and a comprehensive study designed to answer this is needed. We observed that NLRP3 creates a permissive environment for *T. crassiceps* establishment, in which the lack of PDL1^+^PDL2^+^ macrophages was evident, suggesting that the presence of NLRP3 is indispensable for this parasite. Information about the role of NLRP3 in peritoneal macrophage infiltration is scarce, nonetheless, it has been shown that mice lacking NLRP3 are highly resistant to several models of peritoneal inflammation induced by cecal ligation and puncture (CLP) [34], as well as ip. delivery of cholesterol crystals [35], and this resistance was associated with the inability to recruit neutrophils in mice lacking NLRP3. Also, peritoneal fibrosis evoked by methyl-glyoxal (MGO) delivery was attenuated in ASC deficient mice (an adaptor molecule downstream NLRP3), and reduced macrophage infiltration was observed [36]. Peritoneal dynamics seems to be selectively modulated by NLRP3 as evidenced in mouse models of sepsis, where adult mice lacking NLRP3 were protected when induced with polymicrobial sepsis [34], whereas NLRP3 deficiency did not improve survival in neonatal mice underwent cecal slurry [37]. Thus, our data add to the previous evidence showing that NLRP3 contributes to mobilizing innate cells into the peritoneal cavity when a challenge is encountered. Dissecting the mechanism underlying this would be valuable to target NLRP3 to modulate peritoneal infiltration of innate cells.

When both WT and NLRP3^−/−^ mice were infected with *T. crassiceps*, NLRP3^−/−^ mice showed a greater resistance than their WT counterparts (Fig. 2), in line with findings reported for nematode experimental infections where worm burdens of *Trichuris muris* [18], and *Nippostrongylus brasiliensis* [19] were lower in NLRP3^−/−^ mice. Likewise, NLRP3^−/−^ mice were more resistant to *Schistosoma mansoni* experimental infection as gauged by the presence of smaller liver granulomas than those found in WT animals [38]. However, there is an important difference in terms of the mechanisms driving worm clearance among *T. crassiceps* and these nematodes. While nematode expulsion was associated with a strong granulocyte response (eosinophils) along with intestinal Goblet cell hyperplasia and increased alveolar AAMs, we found a progressive decrease in eosinophils and inability to generate AAMs in our cestode infection model (Fig. 3). Furthermore, nematode expulsion was also related to increased IL-4 levels and increased IL-4^+^ T cell differentiation; in contrast, in our hands NLRP3 was relevant in producing IL-4 (Fig. 4), consistent with previous reports where NLRP3 was required, but no other inflammasome components for Th2 polarization and IL-4 release [32]. Due to an intense IL-4 response, macrophages acquire AAMs rewiring which in nematode infections act in concert with peristalsis to ultimately expel worm parasites. Conversely, AAMs enable *T. crassiceps* a successful host colonization by creating systemic suppressive environment, thus, whether AAMs are present dictate disease outcome in this model. Surprisingly, NLRP3^−/−^ mice were virtually not able to recruit macrophages as compared to WT mice which presented increasing population of PDL1^+^ PDL2^+^ macrophages. It has previously been reported that NLRP3 promotes M2 polarization in asthma models [39] and is able to induce PDL1 in lymphoma [40], and supporting this, our data showed that *T. crassiceps* dramatically relies on NLRP3 to recruit macrophages and reprogram them. The latter was supported by our in vitro data demonstrating that at least at transcriptional level, NLRP3 is inducing PDL1 and PDL2 in response to TcES + IL-4 (Fig. 5). Importantly, previous studies have shown that pleural cavity-infiltrating macrophages display a superior ability to acquire PDL2 expression when compared to resident macrophages, and this latter seems to be a limiting step for *Litomosoides sigmodontis* larvae to grow, since this macrophage influx and PDL2 acquisition did not occur in naturally resistant strain [41, 42]. In line with this, during *T. crassiceps* infection, blood derived Ly6C^+^ macrophages have been shown to selectively upregulate PDL2 and exert highly suppressive features [43].

Thus, the importance of a dynamic macrophage peritoneal recruitment to acquire PDL2 expression promoting *T. crassiceps* successful infection, might largely be supported by NLRP3 activity.

As previously mentioned, NLRP3 acts as a transcription factor in T cells activating a Th2 program, thus, whether NLRP3 also displays the ability to directly activate downstream genes such as PDL1 and PDL2 in macrophages under distinct contexts, as our findings suggest, is still a possibility to explore. Intriguingly, when TcES + IL-14 were ip. given, we did not find neither PDL2 expression nor differences between WT and NLRP3^−/−^ mice, suggesting that the role of NLRP3 may be macrophage source-specific, this is a strong possibility given differences in NLRP3 activity between BMMs, and peritoneal macrophages have been reported [44].

Therefore, it remains to be determined how NLRP3 modulates apparently contrasting immune mechanisms (Th2 suppression versus Th2 promotion) leading to the same result, which is worm clearance. This latter could be explained by several posibilities such as differences in parasite life cycle, differential NLRP3 activation on different anatomical sites/sources as well as different helminthic secreted antigens. Further, unlike migrating and intestinal nematodes, *T. crassiceps* does not induce tissue damage which reduces DAMPs release, thus migrating worms would be triggering signaling pathways involving worm-derived PAMPs and tissue injury-derived DAMPs, unlike *T. crassiceps*.

Furthermore, whether NLRP3 inflammasome assembling is required during in vivo infection with *T. crassiceps* must properly be studied, since we found that both IL-1β and IL-18 are differentially released during *T. crassiceps* infection. Whereas circulating IL-18 levels were low and showed a decreasing trend without difference between WT and NLRP3^−/−^ mice, levels of IL-1β progressively increased in *T. crassiceps*-infected WT mice reaching a significant increase in week 8 of infection as compared to NLRP3^−/−^ mice (Fig. 4). Interestingly, NLRP3-independent release of IL-1β has been described [45], including other inflammasomes. Thus, there exists the possibility that NLRP3 inflammasome is indeed assembled intentionally or in a by-stander way as previously described in APCs or additional caspase1-activating inflammasomes are also being activated. Interestingly, IL-1β levels peaked when parasites successfully established which is attributed a global immunosuppression state in host along an overwhelming Th2 response. Also, in vitro BMM transcriptional analysis revealed that TcES is not inducing NLRP3 inflammasome components. Thus, unless a first hit (priming) of macrophages before TcES exposure is required to induce inflammasome activation in response to TcES, our data suggest that at least in BMMs *Taenia* antigens do not harbor molecules to canonically activate NLRP3 inflammasome. Hence, low levels of IL-18 and late induction of IL-1β suggest that *T. crassiceps* might be activating NLRP3 to display features that not necessarily imply IL-1β and IL-18 maturation and release. In fact, Ritter et al. showed that helminth-derived antigens evoked a regulatory program in bone-marrow-derived DCs (BMDCs) while inducing IL-1β in a by-stander manner [38]. This is something we cannot rule out to occur in macrophages exposed to TcES, but convincing evidence must be provided.

Furthermore, in experimental infection with *T. crassiceps* metacestodes (non-migrating larvae in peritoneal cavity), a close relationship with intestinal microbiota is not expected; however, we demonstrated that intestinal microbiota might indeed be able to promote resistance against this parasite. Our co-housing experiments suggest that NLRP3 deficiency results in intestinal dysbiosis wherein, yet to identify, microbial communities influence the immune response impairing AAMs’ appearance (Fig. 6). Of note, co-housing experiments showed a dominant effect of NLRP3^−/−^ mice over WT littermates rather than a bidirectional influence since NLRP3^−/−^ mice showed negligible changes in parasite resistance and macrophages and eosinophil abundance (Fig. 6). Ongoing studies are undertaken to identify bacterial and/or viral communities putatively responsible to control *Taenia* growth.

Thus, our observations indicated that NLRP3 is a pivotal receptor enabling *T. crassiceps* optimal growth, and it is likely that NLRP3 is not directly activated by *Taenia*-derived antigens, rather other molecules emerging during *Taenia*-elicited immune response might be playing a role. Accordingly, *T. crassiceps* infection strongly evokes prostaglandin E2 (PGE2) in AAMs [46], which facilitate host colonization [47], and it was recently shown that PGE2 can activate NLRP3 in peritoneal macrophages during *Staphylococcus infection* [48]. Whether this or other humoral components of the immune response are responsible for NLRP3 activation must be explored.

## Conclusion

Our data demonstrate that endogenous NLRP3 receptor is part of the immune-regulatory network generated by *T. crassiceps* to colonize its host, most likely enabling macrophages to acquire a regulatory program, inducing a Th2 response and controlling T lymphocyte expansion. A critical role for microbiota contributing to protection in *T. crassiceps* infection is strongly suggested as well.

## Supplementary Information

Below is the link to the electronic supplementary material.

Supplementary file1 (PDF 37 KB)

## Data Availability

No datasets were generated or analysed during the current study.
