# Supplementary material for: Endogenous innate sensor NLRP3 is a key component in peritoneal macrophage dynamics required for cestode establishment
Source: Immunol Res. 2024 Jun 6;72(5):948–63. doi: 10.1007/s12026-024-09496-3 (PMC11564225; doi:10.1007/s12026-024-09496-3)
Supplement: Supplementary file 1 — Supplementary file1 (PDF 37 KB) [file 12026_2024_9496_MOESM1_ESM.pdf]

Supplementary table 1

β-actin	Forward GCT GTG CTA TGT TGC TCT AG
	Reverse CGC TCG TTG CCA ATA GTG
pdl1	Forward GCT CCA AAG GAC TTG TAC GTC
	Reverse TGA TCT GAA GGG CAG CAT TTC
pdl2	Forward CTG CCG ATA CTG AAC CTG AGC
	Reverse ACG GTC AAA ATC GCA CTC C
relm α	Forward CCC TTC TCA TCT GCA TCT CC
	Reverse CAG TAG CAG TCA TCC CAG CA
Nlrp3	Forward CAT GAG TGT GGC TAG ATC CAA G
	Reverse ATT ACC CGC CCG AGA AAG G
il1b	Forward TGA GGC CCA AGG CCCA CAG GT
	Reverse AAG CCT CGT GCT GTC GGA CC
il18	Forward GCC TCG GGT ATT CTG TTA TGG A
	Reverse GAC CTG GAA TCA GAC AAC TTT GG
caspase 1	Forward GCA AGA CGT GTA CGA GTG GTT G
	Reverse GGC ACA TTT CCA GGA CTA ACT G
